# Impaired IFN-γ Production by Viral Immunodominant Peptide-specific Tetramer+ CD8+ T Cells in HIV-1 Infected Patients is not Secondary to HAART

**DOI:** 10.1080/17402520400001611

**Published:** 2004

**Authors:** Nattawat Onlamoon, Kovit Pattanapanyasat, Aftab A. Ansari

**Affiliations:** 1 Center of Excellence for Flow Cytometry, Division of Instrument for Research Office for Research and Development Faculty of Medicine, Siriraj Hospital Mahidol University Bangkok Thailand; 2 Department of Pathology and Laboratory Medicine Room 2309 WMB Emory University School of Medicine Atlanta GA USA

## Abstract

Studies on PBMC samples from HIV-1 infected patients have shown that despite substantial number of HIV specific CTLs, these patients gradually progress to AIDS. The present study was conducted to determine whether this paradox was secondary to the influence of protease inhibitors being utilized by these patients. Thus, aliquots of PBMC samples from 10 HIV infected humans with no prior history of anti-retroviral drug therapy (ART) and 6 HIV-infected patients that had been on HAART for >1 year were analyzed for the frequency of HIV-1 Nef and Gag dominant peptide specific tetramer+ cells, respectively. The tetramer+ PBMCs were analyzed for their ability to synthesize specific peptide induced IFN-γ utilizing both the ELISPOT and the intracellular cytokine (ICC) assays. Results of the studies showed that there was an overall correlation between the frequency of Nef and Gag peptide tetramer+ cells and the frequency of IFN-γ synthesizing cells as assayed by either ICC or ELISPOT assay, markedly reduced values of IFN-γ synthesizing cells per unit tetramer+ cells were noted in both group of patients. These data suggest that the frequency of HIV-specific CD8+T cells is maintained during the chronic phase of infection, their ability to function is compromised and is not a reflection of ART. While the addition of IL-2, anti-CD40L and allogeneic cells led to partial increase in the ability of the tetramer+ cells to synthesize IFN-γ, the addition of IL-4, IL-12, anti-CD28 or a cocktail of anti-TGF-β, TNF-α and IL-10 failed to augment the IFN-γ response.


*Clinical & Developmental Immunology*, September/December 2004, Vol. 11 (3/4), pp. 287–298

Taylor & Francis healthsciences

### Impaired IFN-γ Production by Viral Immunodominant Peptide-specific Tetramer+ CD8+ T Cells in HIV-1 Infected Patients is not Secondary to HAART

NATTAWAT ONLAMOON[a], KOVIT PATTANAPANYASAT[a] and AFTAB A. ANSARI[b,*]

[a]*Center of Excellence for Flow Cytometry, Division of Instrument for Research, Office for Research and Development, Faculty of Medicine, Siriraj Hospital, Mahidol University, Bangkok, Thailand;* [b]*Department of Pathology and Laboratory Medicine, Room 2309 WMB, Emory University School of Medicine, 1639 Pierce Drive, Atlanta, GA 30322, USA*

Studies on PBMC samples from HIV-1 infected patients have shown that despite substantial number of HIV specific CTLs, these patients gradually progress to AIDS. The present study was conducted to determine whether this paradox was secondary to the influence of protease inhibitors being utilized by these patients. Thus, aliquots of PBMC samples from 10 HIV infected humans with no prior history of anti-retroviral drug therapy (ART) and 6 HIV-infected patients that had been on HAART for $>1$ year were analyzed for the frequency of HIV-1 Nef and Gag dominant peptide specific tetramer+ cells, respectively. The tetramer+ PBMCs were analyzed for their ability to synthesize specific peptide induced IFN-γ utilizing both the ELISPOT and the intracellular cytokine (ICC) assays. Results of the studies showed that there was an overall correlation between the frequency of Nef and Gag peptide tetramer+ cells and the frequency of IFN-γ synthesizing cells as assayed by either ICC or ELISPOT assay, markedly reduced values of IFN-γ synthesizing cells per unit tetramer+ cells were noted in both group of patients. These data suggest that the frequency of HIV-specific CD8+ T cells is maintained during the chronic phase of infection, their ability to function is compromised and is not a reflection of ART. While the addition of IL-2, anti-CD40L and allogeneic cells led to partial increase in the ability of the tetramer+ cells to synthesize IFN-γ, the addition of IL-4, IL-12, anti-CD28 or a cocktail of anti-TGF-β, TNF-α and IL-10 failed to augment the IFN-γ response.

*Keywords*: CTL; Tetramer; Intracellular cytokine; ELISPOT; Impaired function

### INTRODUCTION

It is generally accepted that control of viremia in HIV-1 infected individuals is mediated to a large part by virus specific cytotoxic CD8+ T effector cells (Koup *et al.*, 1994; Klein *et al.*, 1995). This important role for CD8+ CTLs is supported by results of a number of studies. These studies include the finding that there is a strong correlation between the level of decline in plasma viremia and the level of virus specific CD8+ CTL function during the acute viremia period (Borrow *et al.*, 1994; Koup *et al.*, 1994). Secondly, it is clear that while progression to disease in HIV-1 infected patients is accompanied by loss of virus specific CTL function (Carmichael *et al.*, 1993; Rinaldo *et al.*, 1995), induction and maintenance of strong virus specific CTL function is one of the hallmarks of HIV-1 infected patients who are classified as long term non-progressors (Klein *et al.*, 1995; Harrer *et al.*, 1996a,b). Further support for a prominent role for virus specific CD8+ effector CTLs came from the finding that select individuals who were highly exposed to HIV-1 but had undetectable levels of virus in their blood, appeared to demonstrate a broad HIV-1 specific CTL effector cell population in their peripheral blood mononuclear cells (PBMCs) (Rowland-Jones *et al.*, 1995; Fowke *et al.*, 1996). Unequivocal evidence for an important role that CD8+ effector T cells play in lentiviral infection came from the finding that depletion of this cell lineage in SIV infected rhesus macaques *in vivo* with the use of a depleting monoclonal anti-CD8 antibody, led to marked increases in viral loads associated with progression to disease (Schmitz *et al.*, 1999; Jin *et al.*, 1999).

A prominent role for CD8+ CTL effector function in the clearance of a number of other viral infections in both humans and a variety of experimentally infected animals has long been established (Riddell *et al.*, 1992; Guidotti *et al.*, 1996; Chisari, 1997). To a large extent, such effector CTL function has been measured by conventional bulk functional *in vitro* re-priming CTL assays in which

*Corresponding author. Tel.: +1-404-712-2834. E-mail: pathaaa@emory.edu

ISSN 1740-2522 print/ISSN 1740-2530 online © 2004 Taylor & Francis Ltd
DOI: 10.1080/17402520400001611

either enriched populations of effector CD8+ T cells or CD8+ T cell containing PBMCs were co-cultured in varying ratios with the appropriate virus infected radioactively labeled autologous target cells. Release of radioactivity was utilized as a measure of lytic activity of the effector CD8+ cytotoxic T cell in such assays and the ratio of effector: target cell utilized as a relative assessment of the frequency of viral antigen specific CD8+ T cells (Walker *et al.*, 1987). Use of limiting dilution assays accompanied by use of specific virally encoded synthetic peptides to pulse the target cells provided a somewhat better assessment of the precursor frequency of viral antigen specific effector CD8+ T cells (Carmichael *et al.*, 1993; Koup *et al.*, 1994). These type of assays, however, did not provide a clear picture on the true frequency of peptide specific effector CD8+ T cells since such assays were designed to measure functional activity. An important and major scientific breakthrough was achieved by the seminal findings from the laboratory of Dr Mark Davis with the discovery that specific peptide bearing fluorescently tagged tetrameric conjugates of recombinant MHC class I molecules could be utilized as probes for the identification of peptide specific MHC class I restricted CD8+ T cells by flow cytometry (Altman *et al.*, 1996). The advent of this technology was soon followed by its use for enumerating the frequency of HIV-1 peptide specific MHC class I restricted CD8+ CTL and the finding that there was an inverse correlation between the frequency of such HIV-1 peptide-tetramer binding CD8+ T cells and the level of plasma viremia (Ogg *et al.*, 1998). Of importance was the finding that the frequency of such peptide-tetramer binding CD8+ T cells correlated well with the *in vitro* cytotoxicity assay. These last two pieces of evidence confirmed the previously held view of an important role for CD8+ CTL in the control of viremia in human HIV-1 infected individuals. While tetramer technology paved the way for the precise enumeration of antigen specific T cells, further advances were being made on methodologies to assess antigen specific T cell function by the measurement of cytokine synthesis upon exposure of the T cell receptor to its cognate peptide bearing MHC ligand. These include the ELISPOT assay (Lalvani *et al.*, 1997) and the intracellular synthesis of intracellular cytokines (ICC) such as IFN-γ by CD8+ T cells as a correlate of antigen specific effector function (Betts *et al.*, 2000). These ELISPOT and ICC technologies were soon used in conjunction with the tetramer analysis and these are the assays that continue to be most often utilized for the measurement of antigen specific T cell function (Appay *et al.*, 2000; Goepfert *et al.*, 2000; Goulder *et al.*, 2000).

Results with the use of such assays in PBMCs samples from HIV-1 infected individuals showed that there was a progressive decline in HIV-1 specific peptide-tetramer+ cells as patients developed clinical disease (Rinaldo *et al.*, 2000). Since these studies, a number of additional studies utilizing similar tetramer technology and ICC analysis have provided conflicting data on the frequency of such HIV specific tetramer+ cells and function (Appay *et al.*, 2000; Shankar *et al.*, 2000; Kostense *et al.*, 2001). Thus, while some studies reported that most HIV specific tetramer+ CTLs synthesize IFN-γ but had relatively low levels of perforin (Appay *et al.*, 2000), others documented that $<25\%$ of the HIV specific tetramer+ cells synthesized IFN-γ (Shankar *et al.*, 2000). The low frequency of IFN-γ synthesizing tetramer+ cells could be increased by the supplementation of the culture media with interleukin-2 (Shankar *et al.*, 2000). Each of these studies, however, utilized PBMCs samples from patients who were undergoing various chemotherapeutic regimens including patients who were being administered protease inhibitors that besides their anti-viral effect could also potentially influence assays that involve antigen processing and presentation and thus influence the outcome of the studies (Andre *et al.*, 1998). The present study was therefore undertaken utilizing PBMC samples from patients with no previous history of anti-retroviral drug therapy (ART) and for comparison PBMCs from patients with $>1$ year history of HAART to address this issue. Results of these studies constitute the basis of this report.

### MATERIALS AND METHODS

### Study Population

The studies performed herein were approved by the Ethics Committees, Faculty of Medicine Siriraj Hospital, Mahidol University, Bangkok, Thailand and Emory University School of Medicine, Atlanta, GA. Each study volunteer was explained the nature of the study and appropriate informed consent was obtained prior to the enrollment of the patients in the study. Peripheral blood samples were obtained from a total of 11 non-HIV infected and 24 stage 1 (WHO nomenclature) HIV-1 infected patients in Thailand and 6 archived HLA-A*0201 HIV-1 infected PBMC samples from patients who had been on ART for $>1$ year at the Emory University. An aliquot of these cryopreserved PBMCs were subjected to molecular MHC class I typing (Tissue Typing Laboratory, Emory University Hospital, Atlanta, GA). Four of the 11 non-HIV infected control and 10 of the 24 HIV-1 infected patient samples were found to express the HLA-A*1101 MHC class I allele. None of these 10 patients received any antiretroviral chemotherapy prior to the study. Routine CBC based absolute lymphocyte counts and T cell subset analysis by flow cytometry were determined on aliquots of a blood sample from each patient (Table I). Blood samples were separated on Ficoll-Hypaque (Histopaque 1077; Sigma, St Louis, MO) gradients to obtain PBMC. Aliquots of the PBMCs were cryopreserved in media containing 20% fetal bovine serum (FBS) with 10% DMSO and stored at $-180°C$ prior to thawing and use in the analysis of the peptide-MHC tetramer staining and ELISPOT assays.

TABLE I Value of CD4+ and CD8+ T cell subset

| Patients | | CD4 count/μl | %CD4 | CD8 count/μl | %CD8 | Ratio |
|---|---|---|---|---|---|---|
| No ART (A11) | P14 | 318 | 10.60 | 1633 | 54.45 | 0.195 |
| | P17 | 241 | 14.20 | 917 | 54.05 | 0.263 |
| | P19 | 277 | 10.55 | 1773 | 67.45 | 0.156 |
| | P24 | 221 | 14.90 | 994 | 67.00 | 0.222 |
| | P27 | 309 | 16.60 | 864 | 46.40 | 0.358 |
| | P33 | 328 | 9.20 | 1802 | 50.60 | 0.182 |
| | P38 | 404 | 13.20 | 2176 | 71.10 | 0.190 |
| | P39 | 178 | 7.60 | 1820 | 77.60 | 0.100 |
| | P43 | 311 | 17.25 | 873 | 48.50 | 0.360 |
| | P46 | 229 | 11.95 | 1212 | 63.30 | 0.190 |
| ART (A2) | P50 | 412 | 17.19 | 1728 | 72.12 | 0.238 |
| | P57 | 506 | 18.53 | 1879 | 68.82 | 0.269 |
| | P59 | 578 | 19.32 | 2124 | 71.01 | 0.272 |
| | P64 | 492 | 26.56 | 1024 | 55.29 | 0.480 |
| | P67 | 502 | 22.41 | 1821 | 70.11 | 0.320 |
| | P79 | 435 | 25.16 | 998 | 57.72 | 0.436 |

**Monoclonal Antibody and Reagent**

The following monoclonal antibodies (mAbs) were commercially obtained and utilized at the concentration recommended by the respective manufacturer in the studies reported herein: PerCP-conj. anti-human CD8 (Becton Dickinson Biosciences, San Jose, CA); APC-conj. anti-human CD3 and FITC-conj. anti-IFN-γ (Coulter, Miami, FL). The anti-human CD28 and CD49d (purified) were purchased from Pharmingen (San Diego, CA) and each used at a final concentration of 1 μg/ml. Brefeldin A (BFA: GolgiPlug™) and Monensin (GolgiStop®) were also obtained from Pharmingen and used at 10 μg/ml. Staphylococcal Enterotoxin B (SEB) was purchased from Sigma and used at 10 μg/ml. The HLA-A*1101-restricted epitope from the Nef protein of HIV-1 clade E (QVPLRTMPYK) and the HLA-A*0201-restricted HIV-1 clade B Gag epitope (SLYNTVATL) were synthesized by standard fluorenylmethoxycarbonyl chemistry by the Microchemical facility, Emory University. The peptides were used at 10 μg/ml.

**Cell Surface Staining with Peptide-MHC Tetrameric Complex**

Peptide-MHC tetrameric complex can be used to directly detect antigen specific T cells by flow cytometry (Altman *et al.*, 1996). The HLA A*1101 immuno-dominant Nef peptide "QVPLRTMPYK" and the HLA-A*0201 dominant HIV-1 Gag peptide "SLYNTVATL" were utilized to prepare the tetrameric complex (heretofore referred to as A11Nef and A2Gag) formed by streptavidin conjugated with phycoerythrin (PE) under the supervision of Dr Nattawan Promadej (Division of HIV/AIDS, Centers for Disease Control and Prevention, Atlanta, GA). Briefly, recombinant MHC class I heavy chains isolated from HLA-A 1101 and HLA-A0201 individuals were refolded with the appropriate peptides and $\beta_2$-microglobulin to form peptide-MHC tetrameric complexes. The MHC heavy chains were engineered to contain a biotinylation site at the COOH terminal. The monomer constructs were utilized to form tetrameric complexes by the addition of streptavidin conjugated PE. Aliquots of the cryopreserved PBMCs were thawed, washed two times at 500*g* for 5 min each in RPMI medium (RPMI 1640) containing 10% heat inactivated fetal bovine serum, 1% penicillin–streptomycin and 2 mM L-glutamine (all reagents were purchased from GIBCO, Grand Island, NY). The cells were then re-suspended in medium at $1 \times 10^6$ cells/ml and incubated with the PE-conj. A11nef or A2Gag tetrameric complex, anti-human CD8 PerCP and anti-human CD3 APC mAbs for 30 min at 4°C. Finally, the sample was washed using washing buffer (PBS with 2% fetal bovine serum) and fixed in 1% paraformaldehyde in PBS and stored at 4°C until flow cytometric analysis. The percentage of A11Nef or A2Gag tetramer+ cells among the CD3+ CD8+ T cells population were determined on a FACSCaliber flow cytometer (BDB) using Cellquest software. Data from at least 200,000 cells gated on the lymphocyte population were acquired and subjected to analyses.

**Enumeration of IFN-γ Synthesizing Cells by the ELISPOT Assay**

CTL-M200 (Cellular Technology; Cleveland, OH) 96-well plates were coated overnight at 4°C with 50 μl of anti-IFN-γ mAbs, clone 1-D1K, (Mabtech: Stockholm, Sweden) at 5 μg/ml. The antibody-coated plates were washed four times with PBS and blocked with 100 μl of RPMI medium (RPMI 1640) containing 10% heat inactivated fetal bovine serum, 1% penicillin–streptomycin and 2 mM L-glutamine for 1 h at 37°C. The appropriate PBMCs at $2 \times 10^5$ cells in 200 μl medium were added to duplicate wells and incubated for 36 h at 37°C/5%$CO_2$ with either the HLA-A*1101 restricted Nef peptide or the HLA-A*0201 restricted Gag peptide described above (10 μg/ml). PBMC incubated with PHA (2 μg/ml) were used as a positive control and cells without any peptide or PHA were used as a negative control. The plates were then washed four times with washing buffer consisting of PBS containing 0.05% Tween-20 (Sigma).

To each well, 50 μl of a 1 ug/ml of biotin conjugated anti-IFN-γ mAbs, clone 7-B6-1, (Mabtech, Nacka, Sweden) in PBS containing 1% fetal bovine serum and 0.05% Tween-20 was added. After incubation for 3 h at 37°C/5%$CO_2$, the plates were washed with washing buffer as above and 50 μl of streptavidin conjugated alkaline phosphatase (Mabtech) was added at a dilution of 1:1000 in PBS containing 1% fetal bovine serum and 0.05% Tween-20. This was followed by 1 h incubation at 37°C/5%$CO_2$. The plates were washed again with washing buffer and 50 μl of 1-Step NBT/BCIP (Pierce Chemicals, Rockford, IL) was then added to each well. The reaction was stopped by washing three times with sterile water. The number of spots was enumerated utilizing the Immunospot analyzer (Cellular technology). The number of spots per $10^6$ cells was calculated from the average of duplicate wells subtracting the average spots obtained with the negative control run in parallel.

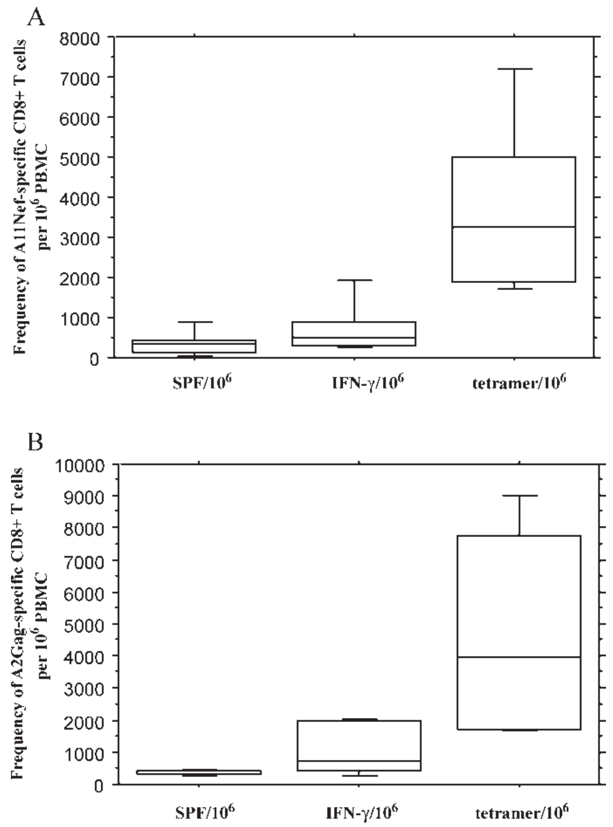

FIGURE 1 Comparison between the number of spot forming unit per $10^6$ PBMC (SPF/$10^6$) by ELISPOT, IFN-γ producing CD3+ CD8+ T cells per $10^6$ PBMC by ICS and the frequency of A11Nef tetramer+ cells per $10^6$ PBMC from ART naïve patient (A) and A2Gag tetramer+ cells from ART treated patient (B).

### Antigen Stimulation and ICC Staining of Tetramer+ Cells

PBMC at $1 \times 10^6$ cells/ml were pre-stained with the tetrameric complex and incubated in a 5 ml polypropylene tube. The cultures were incubated upright for 30 min at 37°C/5%$CO_2$ followed by washing in medium. The cells were resuspended in medium and stimulated with 10 μg/ml of the same HIV peptide as used for the formation of the tetrameric complex in the presence of anti-human CD28 and CD49d mAbs (each at 1 μg/ml) for 2 h at 37°C/5%$CO_2$. PBMCs stimulated with SEB (10 μg/ml) were used as a positive control and cells without any peptide or SEB were used as a negative control. Both BFA and monensin (10 ug/ml) were added for the final 4 h. After the total 6 h of incubation, 100 μl of 20 mM EDTA in PBS was added to each of the stimulated and unstimulated samples and the samples incubated in the dark for 15 min at room temperature (RT). Samples were then fixed in 2 ml of 1 × FACS™ Lysing Solution (BDIS) and incubated in the dark for 10 min at RT followed by centrifugation at 500 *g* for 5 min. Cells were washed in washing buffer and permeabilized with 0.5 ml of 1 × FACS™ Permeabilizing Solution (BDIS) for 10 min at RT in the dark. After permeabilization, cells were washed by adding 2 ml of washing buffer and centrifugation at 500*g* for 5 min. Cell surface and intracellular staining was performed following the addition of an antibody cocktail consisting of anti-human CD8 PerCP, CD3 APC and anti-IFN-γ FITC followed by incubation in the dark for 30 min at RT. After staining, the cells were washed and fixed in 1% paraformaldehyde in PBS pH 7.4 and stored at 4°C until flow cytometric analysis were performed.

### Flow Cytometric Analysis of ICC Production

Six-parameter analysis of the above stained samples was performed on a FACSCaliber flow cytometer (BDB) using Cellquest software. Data from at least 200,000 cells gated on the lymphocyte population were acquired and a lymphogate was done to include only viable lymphocytes. Data were displayed as two-color dot plot (FITC vs PE) to measure the percentage of the double positive (IFN-γ + / tetramer+ ) cells within the CD3+ CD8+ T cells population (upper right quadrant, see Fig. 1 for details). An unstimulated control was used to set the quadrant gate. The percentage of IFN-γ sysnthesizing tetramer+ T cells was calculated within the CD3+ CD8+ tetramer+ T cells population.

### Reconstitution Experiments

PBMCs samples only from the select HLA-A*1101 HIV-1 infected individuals were utilized for these studies. The PBMCs ($1 \times 10^6$/ml) were stained with the A11Nef tetramer as described above and then cultured in the presence or absence of either recombinant human IL-2 (10 U/ml, courtesy Hoffman LaRoche, Nutley, N.J.), anti-CD40L (10 μg/ml, B–D Pharmingen, San Jose, CA), allogeneic irradiated PBMC from a healthy human volunteer (5000 rads, $1 \times 10^6$/ml), human recombinant IL-4 (10 U/ml, Biosource Int., Camarillo, CA), recombinant human IL-12 (Biosource Int., Camarillo, CA), or a cocktail of anti-TGF-β, anti-TNF-α and anti-IL-10

(each at 10 μg/ml, B–D Pharmingen, San Jose, CA) and with or without the Nef peptide (10 μg/ml). Following incubation for 2 h, both BFA and monensin were added (10 μg/ml) for the last 4 h. The cultures were washed and the frequency of A11nef tetramer+ cells synthesizing IFN-γ determined using flow cytofluorometry as described above. A minimum of 200,000 cells was analyzed to calculate the frequency of IFN-γ synthesizing cells. Results are expressed as the net frequency of Nef peptide specific IFN-γ synthesizing cells by deducting the values obtained from cultures incubated without the Nef peptide from the ones incubated with the Nef peptide and the identical reconstituting agents.

To study cytokine production ability after CD4+ T cells depletion, PBMCs from 2 HIV-1 infected HLA-A*1101 patients were subjected to depletion of CD4+ T cells using anti-CD4 coated immunobeads (Dynal Corp., Lake Success, NY) at a ratio of 4 beads per CD4+ T cells. Following depletion, the cells were washed and utilized for the analysis of A11Nef tetramer+ CD3+ CD8+ T cells that synthesized IFN-γ as outlined above.

### Statistical Analysis

The Pearson correlation coefficient test was used to analyze for the association observed between different parameters with a value of $p < 0.05$ being considered significant.

### RESULTS

### Frequency Analysis of HIV-1 Specific CD8+ T Cells as Determined by Tetramer Analysis

In the present study, the A11Nef tetramer reagent was utilized to determine the frequency of A11Nef tetramer+ cells among the CD3+ CD8+ T cell sub-population in the PBMCs from 4 control non-HIV infected and 10 retroviral drug naïve HIV-1 infected patients and the A2Gag tetramer reagent in the same subset of PBMCs from 6 HIV-1 infected patients with a history of ART for >1 year. The frequency of tetramer-binding cells in the 4 control non-HIV infected HLA-A*1101 individuals was <0.03% (data not shown) and ranged from 0.3 to 1.52% (0.77 ± 0.37, mean ± SD) in PBMCs samples from the 10 HIV-1 infected patients (Table II). PBMCs from three of these 10 patients showed a frequency of A11Nef tetramer-binding cells of 1% or greater. In confirmation with previous studies (Ogg *et al.*, 1998), the results showed a negative correlation with absolute CD4+ T cell count ($R = 0.927$, $p = 0.0001$) (data not shown). These data indicate that the loss of CD4+ T cells is associated with an increase in the frequency of HIV-1 specific CD8+ T cells. The frequency of A2Gag tetramer+ cells in the PBMCs samples from the HIV-1 infected patients on ART for >1 year ranged from 0.22 to 1.79 (0.89 ± 0.62, mean ± SD). In contrast, a positive correlation with absolute CD4+ T cell count was observed in these patients. This correlation was not statistically significant ($R = 0.794$, $p = 0.0592$) (data not shown). However, when the results from the patient with low frequency of A2Gag tetramer+ cells (P67) were excluded from the analysis, the correlation became significant ($R = 0.986$, $p = 0.0021$) (data not shown). There was no statistical difference in the frequency of tetramer+ cells in the HIV infected ART drug naïve vs those with a history of >1 year of ART.

### Frequency of HIV-1 Specific Peptide-MHC Tetramer-binding Cells Correlated with Cytokine-producing Cells as Determined by ELISPOT Assay

The functional activity of antigen specific T cells can be determined at a single cell level by the ability of the cells to synthesize select cytokines such as IFN-γ *in vitro* by the ELISPOT assay (Lalvani *et al.*, 1997). In this study, the same A11Nef and the A2Gag restricted peptides as used

TABLE II Comparison analysis of the HIV peptide specific response as determined by tetramer staining, intracellular cytokine staining and ELISPOT

| Patients | | %Tetramer+ in CD3+CD8+ | %IFN-gamma+ in CD3+CD8+ | %IFN-gamma+ in tetramer+ | No. of tetramer/$1 \times 10^6$ | No. of IFN-gamma/$1 \times 10^6$ | SPF/$1 \times 10^6$ |
|---|---|---|---|---|---|---|---|
| No ART (A11) | P14 | 0.43 | 0.11 | 16.11 | 1892 | 501 | 125 |
| | P17 | 1.00 | 0.12 | 11.80 | 4253 | 469 | 108 |
| | P19 | 0.62 | 0.13 | 20.14 | 3711 | 776 | 383 |
| | P24 | 0.91 | 0.17 | 24.57 | 5122 | 887 | 433 |
| | P27 | 0.72 | 0.06 | 10.78 | 2807 | 269 | 333 |
| | P33 | 0.50 | 0.03 | 10.88 | 2249 | 201 | 323 |
| | P38 | 0.30 | 0.05 | 17.74 | 1772 | 281 | 80 |
| | P39 | 1.52 | 0.35 | 32.84 | 9293 | 2379 | 935 |
| | P43 | 0.61 | 0.14 | 14.12 | 1658 | 357 | 38 |
| | P46 | 1.13 | 0.32 | 27.44 | 4994 | 1483 | 790 |
| ART (A2) | P50 | 0.22 | 0.05 | 15.33 | 1667 | 536 | 327 |
| | P57 | 1.32 | 0.31 | 28.92 | 7758 | 2011 | 452 |
| | P59 | 1.79 | 0.34 | 34.56 | 9122 | 1956 | 259 |
| | P64 | 1.11 | 0.24 | 25.58 | 5676 | 922 | 314 |
| | P67 | 0.27 | 0.06 | 18.43 | 2242 | 256 | 426 |
| | P79 | 0.64 | 0.12 | 13.95 | 1723 | 412 | 339 |

for the preparation of the tetramer complexes were used to stimulate A11Nef and A2Gag specific T cells, respectively, from the 2 groups of patients. The results were expressed as spot forming cells (SPF) per 1 million cells (SPF/$10^6$) of PBMCs. As seen in Table II, the frequency of SPF/$10^6$ ranged from 38 to 935 (355 ± 303, mean ± SD) in the ART naïve samples and ranged from 49 to 1256 in the PBMCs from the patients on ART (445 ± 494, mean ± SD). The results showed a negative correlation with the absolute CD4+ T cell count ($R = 0.733$, $p = 0.0159$) (data not shown). A positive correlation was observed between the percentage of A11Nef and IFN-γ producing cells by ELISPOT ($R = 0.806$, $p = 0.0049$) (data not shown). No correlation was observed between the A2Gag tetramer-binding cells and IFN-γ producing cells by ELISPOT. This finding suggests that the A11Nef and the A2Gag tetramer+ cells are functional since they can synthesize IFN-γ following recognition of the cognate peptide.

Since the data on the frequency of tetramer binding cells was derived by gating on the CD3+ CD8+ T cell sub-population and the ELISPOT assay included analysis of total unfractionated PBMCs, the frequency of tetramer binding cells among all PBMCs (lymphocyte and monocyte) was used to calculate the number of tetramer+ cells per 1 million PBMC (Goepfert *et al.*, 2000). The number of tetramer+ cells/$10^6$ ranged from 1658 to 9293 (3775 ± 2340, mean ± SD) in the ART naïve and 1667 to 9122 (4698 ± 3285, mean ± SD) in the patients on ART, as shown in Table II. The number of A11Nef tetramer+ cells/$10^6$ also showed a negative correlation with absolute CD4+ T cell count ($R = 0.874$, $p = 0.001$) (data not shown). These data indicate that a large number of tetramer+ PBMCs failed to show functional activity as determined by the ELISPOT assay. The precise mechanism(s) for this dysfunction remains to be determined.

### HIV-1 Specific T Cells Detected by IFN-γ Production was Lower than the Frequency Detected by Tetramer Staining

Although a correlation between tetramer+ cells and IFN-γ producing cells by ELISPOT assay in the PBMC of HIV infected patients was observed, the number of HIV-1 specific T cells estimated by the ELISPOT assay was a log fold lower than the absolute number of HIV-1 specific peptide-MHC tetramer+ cells. This result suggests that most of the HIV-1 specific T cells are functionally inert. To investigate this issue further, an ICC staining technique was utilized to more specifically enumerate and identify the cytokine producing function of the CD3+ CD8+ T cells sub-population. Following incubation, fixation and permeabilization, staining with anti-cytokine mAbs was performed. Data are typically expressed as a frequency of cytokine producing cells within phenotypically defined T cell subsets. This study used the same peptide as used for the ELISPOT assay and for the enumeration of the tetramer+ cells. The frequency of IFN-γ producing CD3+ CD8+ T cells as determined by flow cytometric analysis of aliquots of PBMC from the ART naive HIV infected patients ranged from 0.03 to 0.35% (0.15 ± 0.11%, mean ± SD) and 0.05 to 0.34 (0.19 ± 0.13, mean ± SD) in PBMCs from patients on ART (Table II). The data in ART naive also showed a negative correlation with absolute CD4+ T cell count ($R = 0.808$, $p = 0.0047$) (data not shown) whereas a positive correlation was observed in patients on ART. This correlation was not statistically significant ($R = 0.761$, $p = 0.0788$) (data not shown). However, when the results from the patient with low frequency of A2Gag tetramer+ cells (P67) were excluded from the analysis, the correlation became significant ($R = 0.947$, $p = 0.0146$) (data not shown). Furthermore, the frequency of IFN-γ producing CD3+ CD8+ T cells correlated with the frequency of A11Nef and A2Gag tetramer+ T cells ($R = 0.864$, $p = 0.0013$ and $R = 0.985$, $p = 0.0004$, respectively) (data not shown). The frequency of IFN-γ producing CD3+ CD8+ T cells from the ART naive correlated with the number of SPF/$10^6$ as detected by ELISPOT ($R = 0.831$, $p = 0.0029$). The results indicate a good correlation between the frequency of HIV-1 specific tetramer+ T cells, the number of IFN-γ producing T cells by both the ELISPOT and ICC assays.

In efforts to compare the number of HIV-1 specific T cells by each method, the number of IFN-γ + cells per 1 million PBMCs was calculated from the frequency of IFN-γ producing CD3+ CD8+ T cells. The number of IFN-γ + cells/$10^6$ PBMCs ranged from 201 to 2379 (760 ± 688, mean ± SD) in the ART naïve and 256 to 2011 (1016 ± 782, mean ± SD) in the patients on ART. A positive correlation was observed between the number of IFN-γ + cells/$10^6$ and the number of tetramer+ cells/$10^6$ in both the ART naïve and the patients on ART ($R = 0.930$, $p =< 0.0001$ and $R = 0.952$, $p = 0.0033$, respectively) (data not shown). The number of IFN-γ + cells/$10^6$ correlate with the number of SPF/$10^6$ as detected by ELISPOT in the ART naive ($R = 0.899$, $p = 0.0004$) (data not shown). The number of IFN-γ + cells/$10^6$ in the ART naive also showed a negative correlation with the absolute number of CD4+ T cells ($R = 0.789$, $p = 0.0067$) (data not shown). These results suggest that while the estimated number of HIV-1 specific T cells by the ICC assay was greater than that obtained by the ELISPOT assay, the values obtained by the ICC assay were still lower than the frequency of tetramer+ cells in both group of patients (Fig. 1A and B). These data suggest that a substantial number of tetramer+ T cells may either have a functional disability to produce IFN-γ or that there is a difference in the kinetics of IFN-γ synthesis among the population of tetramer+ cells. Paucity in the number of cells available for analysis prevented us to study the issue of kinetics in detail. However, in separate studies of a similar nature utilizing PBMCs from Mamu-A01 + SIV infected rhesus macaques, which showed a similar decrease of IFN-γ synthesizing CD8+ p11C-M gag peptide tetramer+

cells, we performed a more detailed analysis of the kinetics of IFN-γ synthesis by the immunodominant p11C-M peptide Mamu-A-01 restricted and specific tetramer binding CD8+ T cells. Results of this study (in preparation) failed to demonstrate any meaningful increases in the frequency of IFN-γ synthesizing tetramer+ cells following either a shorter or a more prolonged incubation period providing indirect evidence that our failure to detect IFN-γ synthesis by HIV specific tetramer+ cells is likely not due to kinetic differences.

### Impaired Function of HIV-1 Specific T Cells can be Detected by ICC Staining in Tetramer+ Cells

To directly determine the functionally inert HIV-specific CTL at a single cell level, a combination method of intracellular staining and tetramer staining of tetramer+ cells was developed (Appay *et al.*, 2000). Aliquots of PBMCs from each of the 2 groups of patients (ART naïve and those on ART) were stained using the peptide-tetramer complexes prior to antigen stimulation. The pre-staining of the A11Nef and the A2Gag specific tetramer+ cells was followed by antigenic stimulation with the same peptides as used in the formation of the tetramer reagents for each set of patient samples. IFN-γ synthesis within tetramer+ cells was detected by the ICC assay. Only the flow cytometric profile obtained with the A11Nef samples are presented herein for the sake of brevity. The frequency of IFN-γ producing tetramer+ cells (upper right quadrant, Fig. 2C) in a representative sample is illustrated. As seen, most of the tetramer+ cells could not synthesize IFN-γ. It is possible that some of the IFN-γ producing cells could not be stained by the peptide-tetramer complex possibly due to TCR down modulation following activation (lower right quadrant). To calculate the percentage of IFN-γ producing tetramer+ T cells within the tetramer+ population, only IFN-γ producing cells within the upper right quadrant were used (see Fig. 2D).

The percentage of IFN-γ producing tetramer+ T cells within the CD3+ CD8+ tetramer+ T cells ranged from 10.78 to 32.84 (18.64 ± 7.55, mean ± SD) in the ART naïve patient samples and 13.95 to 34.56 (22.8 ± 8.3, mean ± SD) in the samples from patients on ART. PBMCs from 9/10 and 5/6 patients in the 2 groups showed $<30\%$ frequency of IFN-γ producing tetramer+ T cells. These data indicate that not all tetramer+ cells remain functionally active. The result from the ART naïve and patients on ART showed a positive correlation with the frequency of tetramer-binding cells ($R = 0.692, p = 0.0266$ and $R = 0.930, p = 0.0073$, respectively) (data not shown) and IFN-γ producing cells by the ICC assay ($R = 0.887$, $p = 0.0006$ and $R = 0.934, p = 0.0064$, respectively) (data not shown). Interestingly, a negative correlation was observed between the frequency of IFN-γ producing tetramer+ T cells and absolute CD4+ T cell count in the ART naïve ($R = 0.657$, $p = 0.0392$) (data not shown) whereas a positive correlation with absolute CD4 count was observed in patients on ART ($R = 0.895, p = 0.0159$) (data not shown). This indicates that a significant number of functional CTL exist even in the absence of circulating CD4+ T cells.

### Attempts to Reconstitute the IFN-γ Response of the A11 Nef Tetramer+ CD8+ T Cells

While controversy continues to exist on the quantitative aspects of the frequency of HIV-1 antigen specific CD8+ dysfunctional cells among the viral peptide bearing tetramer+ cells, most if not all these studies have to large extent been performed on patients on a variety of anti-retroviral therapies. Such therapies have included protease inhibitors in some patients not others. It was reasoned that one of the reasons for such discrepant results could be the effect of such anti-viral drugs on the immune parameters being measured, in particular, the effect protease inhibitors would have on antigen processing

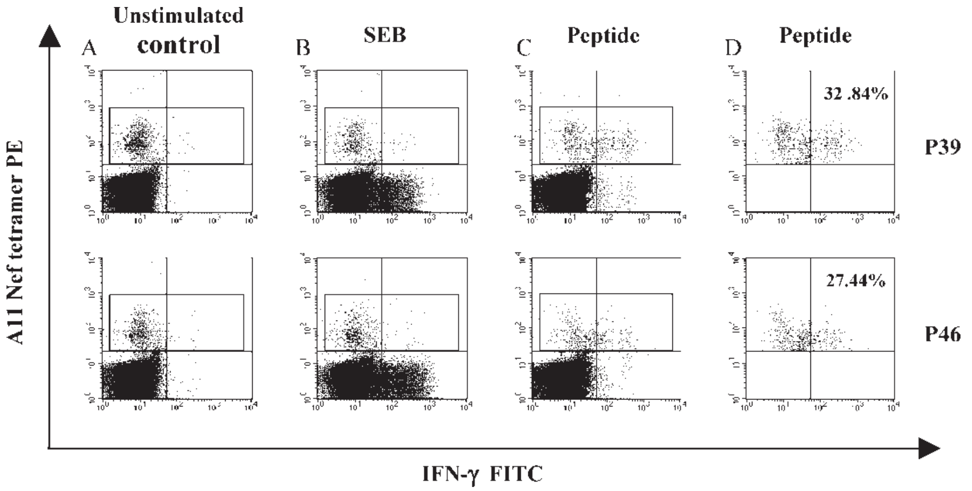

FIGURE 2 Flow cytometric four-colour analysis of CD3+ CD8+ T cell from unstimulated control (A), SEB stimulation (B) and peptide stimulation (C and D). Upper left quadrant (IFN-γ− /tetramer+ ); upper right quadrant (IFN-γ+ / tetramer+ ); lower left quadrant (IFN-γ− /tetramer-); lower right quadrant (IFN-γ+ /tetramer− ). The percentage of IFN-γ producing tetramer+ T cells was calculated within the tetramer+ population (square region) as shown in Fig. 2D.

and presentation. Thus, the present study was undertaken using PBMCs samples from a cohort of HIV-1 infected patients with no prior history of ART. Results of the studies as shown above clearly document the marked decrease in the ability of a significant frequency of the A11nef tetramer+ cells to synthesize IFN-γ. Thus, these results confirm previous findings that document such HIV-1 antigen positive CD8+ T cell dysfunction (Goepfert *et al.*, 2000; Shankar *et al.*, 2000; Kostense *et al.*, 2001). In efforts to delineate potential mechanism(s) that maybe contributing to such dysfunction, a select number of samples ($n = 3$) from the same cohort of HIV-1 infected HLA-A*1101 patients from whom sufficient PBMCs samples could be obtained (P19, P38, P46) were first stained with the same A11nef tetramer reagent and then cultured *in vitro* with the same Nef peptide in the presence or absence of a number of cytokines/agents previously thought to enhance or suppress prototype TH1 like (in this case IFN-γ) immune function and/or antibodies against cytokines thought to suppress TH1 prototype immune function. The enhancing cytokines/agents included IL-2, IL-12, allogeneic irradiated PBMCs and the CD40L stimulating antibody. The suppressing cytokine specific antibodies included anti-TGF-β, TNF-α and IL-10 which were combined and used as a cocktail due to the paucity of the cell sample. As seen in Fig. 3, whereas incubation of aliquots of the PBMCs with IL-2, allogeneic cells and anti-CD40L antibody led to partial immune reconstitution, incubation with IL-4, IL-12 or the cocktail of anti-TGF-β, TNF-α and IL-10 antibodies failed to demonstrate any significant augmenting effect.

Recently, there has been a renewed interest on a potential role of CD4+, CD25+ regulatory T cells in the regulation of immune responses (Shevach *et al.*, 2001). It was thus reasoned that such phenotypic cells could potentially play a role in regulating the response of the A11Nef tetramer+ cells in their ability to synthesize IFN-γ upon challenge with the cognate nef peptide. Unfractionated or CD4 depleted PBMCs from 2 HIV-1 HLA-A*1101 patients were subjected to analysis for A11Nef tetramer+ cells that synthesize IFN-γ using the same technique as described above. Results of these studies in fact showed a decrease in the frequency of A11Nef tetramer+ CD3+ CD8+ cells that could synthesize IFN-γ (26.4 and 27.8% in unfractionated and 18.2 and 12.9%, respectively, in the CD4 depleted cultures). These data, although obtained on only 2 patients, support the view that the dysfunction is likely not due to Treg CD4+ T cells and the presence of CD4+ T cells may be required for optimal HIV-1 peptide specific response by the CD8+ T cells. It is recognized that the role of Treg cells could be better assessed by selective depletion of only the CD4+ CD25+ cells, however, once again, the paucity of cell numbers precluded such experimentation.

### DISCUSSION

A number of studies have been conducted aimed at defining the presence/absence and relative frequency of HIV-1 specific CTLs in patients at varying stages of HIV-1 infection (Carmichael *et al.*, 1993; Rinaldo *et al.*, 1995). There has been a general consensus with regards to some issues and not others. Thus, it is generally accepted that there is a readily recognizable and at times robust HIV-1 specific CTL response during and shortly after the acute infection period (Koup *et al.*, 1994; Borrow *et al.*, 1994). In general, there is also a consensus that there is a gradual loss of HIV-1 specific CTLs with progression to disease and loss of CD4+ T cells (Carmichael *et al.*, 1993; Klein *et al.*, 1995; Rinaldo *et al.*, 1995). Finally, data do support the view that LTNP maintain a readily recognizable and detectable level of HIV-1 specific CTLs population which

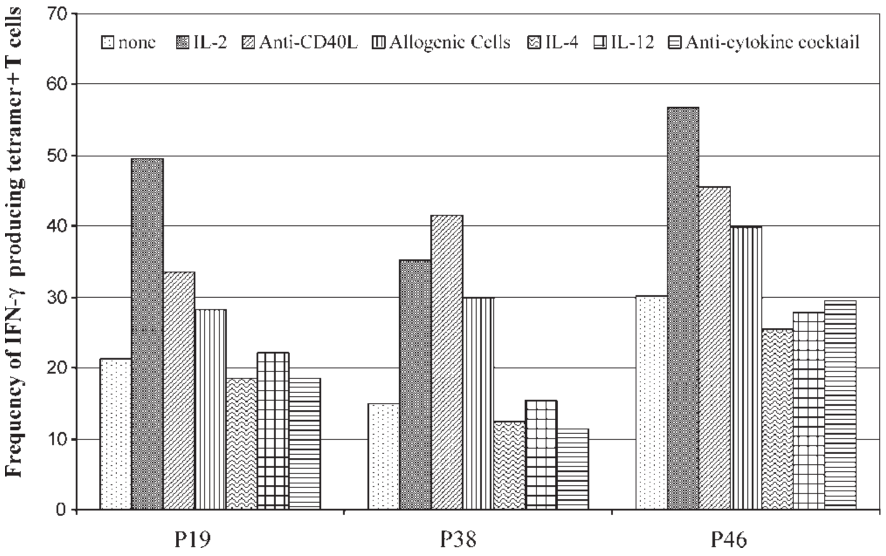

FIGURE 3 Reconstitution of the HIV-1 Nef peptide specific IFN-γ synthesizing response by A11Nef peptide tetramer+ CD8+ T cells from HIV-1 infected patients.

could be contributing to the asymptomatic state of these patients (Klein *et al.*, 1995; Harrer *et al.*, 1996a,b). Whereas a large number of these findings were based on functional CTLs assays, the advent of peptide specific effector cell detection using tetramer technology provided a re-examination of the concepts above. Thus, some studies utilizing immunodominant peptides of either HIV-1 Env, Gag, or Nef to prepare HLA-tetramer reagents to detect CD8+ MHC class I restricted HIV-1 specific CTLs, appeared to suggest that select patients appeared to progress to disease despite the presence of significant numbers of HIV peptide specific tetramer+ cells (Spiegel *et al.*, 2000). Other studies, however, appeared to show a relatively good correlation between the presence of select HIV-1 peptide specific functional HIV specific CTLs and the frequency of the same HIV-1 peptide specific tetramer binding cells (Ogg *et al.*, 1998; Appay *et al.*, 2000; Goulder *et al.*, 2000). The utilization of the peptide specific ICC assay as a correlate of a functionally identical peptide specific CTL assay provided some clues as to the potential reasons for the discrepant results. Thus, it appears that not all peptide tetramer+ cells in the PBMCs of some HIV infected patients synthesize IFN-γ upon incubation with the same specific peptide. One of the explanations provided for these findings was that while the frequency of HIV peptide specific CD8+ T cells are maintained, a large number of them basically become dysfunctional. Since these findings were made on patients with low or undetectable level of plasma viremia, a role for viral load was discounted as a potential reason for these findings. It was also reasoned that these findings could be secondary to the influence of the anti-retroviral drugs that most if not all the patients were taking during the studies performed. Several anti-retroviral drugs specially the protease inhibitors, have been shown to influence immune responses (Andre *et al.*, 1998; Chougnet *et al.*, 2001; Gruber *et al.*, 2001; Stranford *et al.*, 2001) and thus their involvement could be easily envisaged. These thoughts formed the basis for the rationale of the studies performed herein. Thus, PBMCs samples were obtained from the 2 selected groups of HIV-1 infected patients following careful screening of the history of these patients for levels of plasma viral loads and the use of anti-retroviral drugs. Thus, while the plasma viral loads were >10,000 viral copies/ml of plasma in the ART naïve group, the levels were <50 copies/ml of plasma of the patients on ART. The data on the history of anti-retroviral drug use by the drug naive HIV-1 infected patients were reasoned to be highly reliable since the availability of anti-retroviral drugs is highly limited in this study population. Thus, these samples from these 2 groups of patients provided samples that represented patients with relatively high viral loads with no history of ART and patients with low to undetectable levels of plasma viremia and a recorded history of ART.

Several potential mechanisms could be reasoned to be the basis of such impaired function. Thus, this impaired function may due to inappropriate activation of these cells. The down-regulation of CD3ζ and CD28 has been previously observed in HIV-specific CD8+ tetramer+ T cells (Trimble *et al.*, 2000). These molecules play an important role in T cell activation. The loss of these molecules in HIV-specific CTLs may cause a defect by providing insufficient and/or sub-optimal activation signals to produce a potent effector function. Another possible explanation is the loss of help from CD4+ T cells due to the depletion of CD4+ T cells during the chronic phase of viral infection which leads to uncontrolled viral replication even though CTL responses have been shown not to require CD4+ T cells during primary infection in select murine models (Zajac *et al.*, 1998). A study of samples from HIV-1 infected patients showed a positive correlation between HIV-specific CTL precursor frequency and antigen specific CD4+ T cell proliferative response (Kalams *et al.*, 1999). Moreover, another study showed that a loss of IFN-γ producing CTLs correlated with declining CD4+ T cells counts indicating that CD4+ T cells loss in HIV infection may cause CTL dysfunction by the lack of a helper signal for appropriate activation of HIV-specific CTLs (Kostense *et al.*, 2002).

In the studies reported herein, we found a negative correlation between the frequency of IFN-γ producing tetramer+ T cells and absolute CD4+ T cell count in the ART naive patients. These data suggest that even when there is a significant loss of CD4+ T cells during HIV infection, a significant frequency of HIV-specific CTLs are maintained and remains functionally conserved. This result is in agreement with previous studies, which showed a high frequency of HIV and CMV-specific CTLs detected by peptide–tetramer complexes in the absence of circulating peripheral CD4+ T cells (Spiegel *et al.*, 2000). The presence of a significant frequency of HIV-specific CTLs in the recirculating pool of PBMCs may be due to a loss of the ability of such cells to home into infection sites such as lymph nodes, which is secondary to the lack of the expression of lymphoid homing molecules such as CCR7 and CD62L (Chen *et al.*, 2001). However, the precise mechanisms that maintain the existence of such pools of HIV-specific CTLs in the absence of optimal levels of CD4+ T cells remains to be elucidated. In contrast to the ART naïve patients, the results also showed a positive correlation between the frequency of IFN-γ producing tetramer+ T cells and absolute CD4+ T cell count in the patients on ART even though no significant difference of HIV-specific CTLs were observed between these two groups of patients. This result is in agreement with previous studies, which showed the loss of IFN-γ producing tetramer+ T cells correlated with declining CD4+ T cell count (Kostense *et al.*, 2002). The different results observed between these two groups of patients might be due to the effect of ART on the distribution of circulating CD4+ and CD8+ T cell after therapy. However, the relationship between HIV-specific CTLs and CD4+ T cells before and during ART are unclear and remains to be elucidated.

Results of the studies performed herein also confirm the findings of previous studies (Goepfert *et al.*, 2000; Shankar *et al.*, 2000; Kostense *et al.*, 2001). Thus, whereas significant numbers of HIV-1 Nef immunodominant peptide specific CTLs were observed in the PBMCs of these anti-retroviral drug naïve population, the frequency of IFN-γ synthesizing cells were a log lower in absolute value as compared to the absolute values for the same peptide specific tetramer binding cells (see Fig. 1A and Table II). This was also true when one examined the absolute number of IFN-γ synthesizing cells by the tetramer+ CD8+ T cells in these patients, although the ICC assay was a lot more sensitive than the ELISPOT assay giving values which showed a 5-fold decrease by the ICC as compared to 10-fold by the ELISPOT assay. What was not clear from these data was whether these decreased values of HIV-1 specific functional cells is due to an intrinsic reversible/irreversible defect among the CD8+ T cells or that heterogeneity exists among clonal population of HIV-1 peptide specific CTLs. Since the tetramer+ cells express the same relative density of TCR (see Fig. 2D), it is likely that the functional inability is not due to differences in affinity among the tetramer+ cells. These thoughts prompted the preliminary reconstitution studies reported herein.

Attempts were made to determine the potential mechanisms for such dysfunction. First of all, it was reasoned that such dysfunction could merely be a reflection of a chronic viral infection and as such would be manifest for all chronic viral infections. While this issue is difficult to appropriately address in humans, the chronic LCMV infected mice provides a reasonable model to address this issue. As described elsewhere (Welsh, 2001), however, this was not the case since the frequency of IFN-γ synthesizing LCMV specific CD8+ T cells did not decrease during the chronic infection period. Thus, although a more detailed study of a number of other chronic viral infections needs to be performed, particularly in humans, it is possible that the dysfunction noted herein is likely to be secondary to the immunodeficient state of such HIV-1 infected patients. Secondly, it was reasoned that such dysfunction could be secondary to an abnormal cytokine mileu. To address this issue, a study was carried out whereby PBMCs from 3 HLA-A*1101 positive HIV-1 infected patients were cultured with cytokine and/or agents known to augment TH1 prototype immune responses (such as IL-2, IL-12, anti-CD40L, allogeneic cells) and neutralize immune suppressive cytokines (such as TGF-β, TNF-α and IL-10). Results of these studies showed that whereas partial immune reconstitution (herein utilized to signify increase in the frequency of A11Nef tetramer+ cells to synthesize IFN-γ) was noted with the use of IL-2, CD40L antibody and allogeneic cells, such augmented immune responses were not noted with the use of IL-12, IL-4 or a cocktail of anti-TGF-β, TNF-α and IL-10 antibodies. One could argue that the use of a single concentration of the reagents utilized and the short incubation time may not be optimal to observe desired effects. While such a critique is clearly reasonable, with the limited availability of patient sample and the observation of clearly positive augmentation by some of these agents, minimally provides some clues as to the potential mechanisms involved. It is intriguing that whereas anti-CD40L did appear to augment IFN-γ response, IL-12 failed to demonstrate any effect, although signals provided to CD4+ T cells by these agents are both generated by APCs. It is possible that the differences in the signals induced by IL-12 as compared with CD40L ligation could account for the data observed. Since the pathways by which such signaling is mediated is at least partially known, it would be important in the future to further dissect out the molecular mechanisms by which the CD40L induced pathway is functional but not the IL-12. In the latter case a recently described assay for STAT4 and phosphorylated STAT4 would be a reasonable initial approach (Uzel *et al.*, 2001).

It is important to note that none of the antibodies against the putative immune suppressing cytokines appeared to influence the IFN-γ response of the A11nef tetramer+ CD8+ T cells. Although preliminary, these data appear to suggest that there is limited if any role for such cytokines in modulating the IFN-γ response of the antigen specific CD8+ T cells, at least *in vitro*. Finally, the results of the CD4+ T cell depletion prior to analysis of the A11Nef tetramer+ cells to synthesize IFN-γ is of interest. Thus, while a prominent immunoregulatory role for the CD4+ CD25+ Treg cells has been documented in a wide variety of animal models, its role in human immune function remains to be fully elucidated. In the studies reported herein, there does not appear to be a role for such Treg cells. However, it is recognized that results of such an assay need to be interpreted with caution, since removal of all CD4+ T cells could have also eliminated CD4+ T helper function mediated by the few CD4+ T cells remaining in these patients. Specific depletion of the CD4+ CD25+ but not the remainder of the CD4+ T cell pool would have been an ideal for properly interpreting the data. Unfortunately, the restricted number of cells did not permit such a study. Future studies aimed at performing such a study are currently underway. We submit that the cellular and molecular basis of antigen specific CD8+ T cell dysfunction in HIV-1 infection needs to be more fully elucidated to design platforms for full immune reconstitution studies in human HIV-1 infected patients.

In summary, the data presented confirms the previous finding of the presence of a significant frequency of HIV-1 antigen specific dysfunctional CD8+ T cells in the circulation of chronically HIV-1 infected patients. Such dysfunction was not determined to be secondary to either the absence of circulating antigen or due to the use of ART. The mechanisms by which such functionally inactive CD8+ T cells survive for prolonged periods of time remains to be elucidated. Such dysfunction could be partially reconstituted by the exogenous addition of IL-2, allogeneic cells and anti-CD40L but not by IL-12, IL-4 or by the addition of a cocktail of antibodies against TGF-β, TNF-α and IL-10. These data provide some initial insights on the avenues for further studies aimed at delineating the mechanisms of immune dysfunction in HIV-1 infected patients.

### Acknowledgements

The authors gratefully acknowledge the kind co-operation of the HIV and non HIV infected patients that contributed blood samples for the studies reported herein and the primary care physicians who worked hard to provide us the needed samples. Special appreciation also goes to Dr Pilaipan Puthavathana for her kind co-operation in providing us with the blood samples from a cohort study. The authors are grateful to Dr Nattawan Promadej (CDC, Atlanta, GA) for providing help with the preparation of the tetramer reagents and Dr Chris Ibegbu and Dr John D. Altman for providing the principal author with training and access to laboratory facilities to complete some of the studies as outlined herein. Finally, we would like to thank the Royal Golden Jubilee Ph.D. Program, Thailand Research Fund for their financial support to N.O.

### References

Altman, J.D., Moss, P.A., Goulder, P.J., Barouch, D.H., McHeyzer-Williams, M.G., Bell, J.I., *et al.* (1996) "Phenotypic analysis of antigen-specific T lymphocytes", *Science* **274**(5284), 94–96.

Andre, P., Groettrup, M., Klenerman, P., de Giuli, R., Booth, B.L, Jr., Cerundolo, V., *et al.* (1998) "An inhibitor of HIV-1 protease modulates proteasome activity, antigen presentation, and T cell responses", *Proc. Natl Acad. Sci. USA* **95**(22), 13120–13124.

Appay, V., Nixon, D.F., Donahoe, S.M., Gillespie, G.M., Dong, T., King, A., *et al.* (2000) "HIV-specific CD8(+) T cells produce antiviral cytokines but are impaired in cytolytic function", *J. Exp. Med.* **192**(1), 63–75.

Betts, M.R., Casazza, J.P., Patterson, B.A., Waldrop, S., Trigona, W., Fu, T.M., *et al.* (2000) "Putative immunodominant human immunodeficiency virus-specific CD8(+) T-cell responses cannot be predicted by major histocompatibility complex class I haplotype", *J. Virol.* **74**(19), 9144–9151.

Borrow, P., Lewicki, H., Hahn, B.H., Shaw, G.M. and Oldstone, M.B. (1994) "Virus-specific CD8+ cytotoxic T-lymphocyte activity associated with control of viremia in primary human immunodeficiency virus type 1 infection", *J. Virol.* **68**(9), 6103–6110.

Carmichael, A., Jin, X., Sissons, P. and Borysiewicz, L. (1993) "Quantitative analysis of the human immunodeficiency virus type 1 (HIV-1)-specific cytotoxic T lymphocyte (CTL) response at different stages of HIV-1 infection: differential CTL responses to HIV-1 and Epstein–Barr virus in late disease", *J. Exp. Med.* **177**(2), 249–256.

Chen, G., Shankar, P., Lange, C., Valdez, H., Skolnik, P.R., Wu, L., *et al.* (2001) "CD8 T cells specific for human immunodeficiency virus, Epstein-Barr virus, and cytomegalovirus lack molecules for homing to lymphoid sites of infection", *Blood* **98**(1), 156–164.

Chisari, F.V. (1997) "Cytotoxic T cells and viral hepatitis", *J. Clin. Investig.* **99**(7), 1472–1477.

Chougnet, C., Jankelevich, S., Fowke, K., Liewehr, D., Steinberg, S.M., Mueller, B.U., *et al.* (2001) "Long-term protease inhibitor-containing therapy results in limited improvement in T cell function but not restoration of interleukin-12 production in pediatric patients with AIDS", *J. Infect. Dis.* **184**(2), 201–205.

Fowke, K.R., Nagelkerke, N.J., Kimani, J., Simonsen, J.N., Anzala, A.O., Bwayo, J.J., *et al.* (1996) "Resistance to HIV-1 infection among persistently seronegative prostitutes in Nairobi, Kenya", *Lancet* **348**(9038), 1347–1351.

Goepfert, P.A., Bansal, A., Edwards, B.H., Ritter, G.D, Jr., Tellez, I., McPherson, S.A., *et al.* (2000) "A significant number of human immunodeficiency virus epitope-specific cytotoxic T lymphocytes detected by tetramer binding do not produce gamma interferon", *J. Virol.* **74**(21), 10249–10255.

Goulder, P.J., Tang, Y., Brander, C., Betts, M.R., Altfeld, M., Annamalai, K., *et al.* (2000) "Functionally inert HIV-specific cytotoxic T lymphocytes do not play a major role in chronically infected adults and children", *J. Exp. Med.* **192**(12), 1819–1832.

Gruber, A., Wheat, J.C., Kuhen, K.L., Looney, D.J. and Wong-Staal, F. (2001) "Differential effects of HIV-1 protease inhibitors on dendritic cell immunophenotype and function", *J. Biol. Chem.* **276**(51), 47840–47843.

Guidotti, L.G., Ishikawa, T., Hobbs, M.V., Matzke, B., Schreiber, R. and Chisari, F.V. (1996) "Intracellular inactivation of the hepatitis B virus by cytotoxic T lymphocytes", *Immunity* **4**(1), 25–36.

Harrer, T., Harrer, E., Kalams, S.A., Barbosa, P., Trocha, A., Johnson, R.P., *et al.* (1996) "Cytotoxic T lymphocytes in asymptomatic long-term nonprogressing HIV-1 infection. Breadth and specificity of the response and relation to *in vivo* viral quasispecies in a person with prolonged infection and low viral load", *J. Immunol.* **156**(7), 2616–2623.

Harrer, T., Harrer, E., Kalams, S.A., Elbeik, T., Staprans, S.I., Feinberg, M.B., *et al.* (1996) "Strong cytotoxic T cell and weak neutralizing antibody responses in a subset of persons with stable nonprogressing HIV type 1 infection", *AIDS Res. Hum. Retroviruses* **12**(7), 585–592.

Jin, X., Bauer, D.E., Tuttleton, S.E., Lewin, S., Gettie, A., Blanchard, J., *et al.* (1999) "Dramatic rise in plasma viremia after CD8(+) T cell depletion in simian immunodeficiency virus-infected macaques", *J. Exp. Med.* **189**(6), 991–998.

Kalams, S.A., Buchbinder, S.P., Rosenberg, E.S., Billingsley, J.M., Colbert, D.S., Jones, N.G., *et al.* (1999) "Association between virus-specific cytotoxic T-lymphocyte and helper responses in human immunodeficiency virus type 1 infection", *J. Virol.* **73**(8), 6715–6720.

Klein, M.R., van Baalen, C.A., Holwerda, A.M., Kerkhof Garde, S.R., Bende, R.J., Keet, I.P., *et al.* (1995) "Kinetics of Gag-specific cytotoxic T lymphocyte responses during the clinical course of HIV-1 infection: a longitudinal analysis of rapid progressors and long-term asymptomatics", *J. Exp. Med.* **181**(4), 1365–1372.

Kostense, S., Ogg, G.S., Manting, E.H., Gillespie, G., Joling, J., Vandenberghe, K., *et al.* (2001) "High viral burden in the presence of major HIV-specific CD8(+) T cell expansions: evidence for impaired CTL effector function", *Eur. J. Immunol.* **31**(3), 677–686.

Kostense, S., Vandenberghe, K., Joling, J., Van Baarle, D., Nanlohy, N., Manting, E., *et al.* (2002) "Persistent numbers of tetramer$^{+}$ CD8(+) T cells, but loss of interferon-gamma + HIV-specific T cells during progression to AIDS", *Blood* **99**(7), 2505–2511.

Koup, R.A., Safrit, J.T., Cao, Y., Andrews, C.A., McLeod, G., Borkowsky, W., *et al.* (1994) "Temporal association of cellular immune responses with the initial control of viremia in primary human immunodeficiency virus type 1 syndrome", *J. Virol.* **68**(7), 4650–4655.

Lalvani, A., Brookes, R., Hambleton, S., Britton, W.J., Hill, A.V. and McMichael, A.J. (1997) "Rapid effector function in CD8+ memory T cells", *J. Exp. Med.* **186**(6), 859–865.

Ogg, G.S., Jin, X., Bonhoeffer, S., Dunbar, P.R., Nowak, M.A., Monard, S., *et al.* (1998) "Quantitation of HIV-1-specific cytotoxic T lymphocytes and plasma load of viral RNA", *Science* **279**(5359), 2103–2106.

Riddell, S.R., Watanabe, K.S., Goodrich, J.M., Li, C.R., Agha, M.E. and Greenberg, P.D. (1992) "Restoration of viral immunity in immunodeficient humans by the adoptive transfer of T cell clones", *Science* **257**(5067), 238–241.

Rinaldo, C.R, Jr., Beltz, L.A., Huang, X.L., Gupta, P., Fan, Z. and Torpey, 3rd, D.J. (1995) "Anti-HIV type 1 cytotoxic T lymphocyte effector activity and disease progression in the first 8 years of HIV type 1 infection of homosexual men", *AIDS Res. Hum. Retroviruses* **11**(4), 481–489.

Rinaldo, C.R, Jr., Huang, X.L., Fan, Z., Margolick, J.B., Borowski, L., Hoji, A., *et al.* (2000) "Anti-human immunodeficiency virus type 1 (HIV-1) CD8(+) T-lymphocyte reactivity during combination antiretroviral therapy in HIV-1-infected patients with advanced immunodeficiency", *J. Virol.* **74**(9), 4127–4138.

Rowland-Jones, S., Sutton, J., Ariyoshi, K., Dong, T., Gotch, F., McAdam, S., *et al.* (1995) "HIV-specific cytotoxic T-cells in HIV-exposed but uninfected Gambian women", *Nat. Med.* **1**(1), 59–64.

Schmitz, J.E., Kuroda, M.J., Santra, S., Sasseville, V.G., Simon, M.A., Lifton, M.A., *et al.* (1999) "Control of viremia in simian immunodeficiency virus infection by CD8+ lymphocytes", *Science* **283**(5403), 857–860.

Shankar, P., Russo, M., Harnisch, B., Patterson, M., Skolnik, P. and Lieberman, J. (2000) "Impaired function of circulating HIV-specific CD8(+) T cells in chronic human immunodeficiency virus infection", *Blood* **96**(9), 3094–3101.

Shevach, E.M., McHugh, R.S., Piccirillo, C.A. and Thornton, A.M. (2001) "Control of T-cell activation by CD4+ CD25+ suppressor T cells", *Immunol. Rev.* **182**, 58–67.

Spiegel, H.M., Ogg, G.S., DeFalcon, E., Sheehy, M.E., Monard, S., Haslett, P.A., *et al.* (2000) "Human immunodeficiency virus type 1- and cytomegalovirus-specific cytotoxic T lymphocytes can persist at high frequency for prolonged periods in the absence of circulating peripheral CD4(+) T cells", *J. Virol.* **74**(2), 1018–1022.

Stranford, S.A., Ong, J.C., Martinez-Marino, B., Busch, M., Hecht, F.M., Kahn, J., *et al.* (2001) "Reduction in CD8+ cell noncytotoxic anti-HIV activity in individuals receiving highly active antiretroviral therapy during primary infection", *Proc. Natl Acad. Sci. USA* **98**(2), 597–602.

Trimble, L.A., Shankar, P., Patterson, M., Daily, J.P. and Lieberman, J. (2000) "Human immunodeficiency virus-specific circulating CD8 T lymphocytes have down-modulated CD3zeta and CD28, key signaling molecules for T-cell activation", *J. Virol.* **74**(16), 7320–7330.

Uzel, G., Frucht, D.M., Fleisher, T.A. and Holland, S.M. (2001) "Detection of intracellular phosphorylated STAT-4 by flow cytometry", *Clin. Immunol.* **100**(3), 270–276.

Walker, B.D., Chakrabarti, S., Moss, B., Paradis, T.J., Flynn, T., Durno, A.G., *et al.* (1987) "HIV-specific cytotoxic T lymphocytes in seropositive individuals", *Nature* **328**(6128), 345–348.

Welsh, R.M. (2001) "Assessing CD8 T cell number and dysfunction in the presence of antigen", *J. Exp. Med.* **193**(5), F19–F22.

Zajac, A.J., Blattman, J.N., Murali-Krishna, K., Sourdive, D.J., Suresh, M., Altman, J.D., *et al.* (1998) "Viral immune evasion due to persistence of activated T cells without effector function", *J. Exp. Med.* **188**(12), 2205–2213.

